# Japanese health utilities index mark 3 (HUI3): measurement properties in a community sample

**DOI:** 10.1186/s41687-020-0175-5

**Published:** 2020-01-29

**Authors:** Shinichi Noto, Takamoto Uemura

**Affiliations:** 1 Department of Rehabilitation, Niigata University of Health and Welfare, 1398 Shimami, Kita-ku, Niigata city, Niigata, Japan; 2 A Corporate Juridical Person, Life Science Promotion Association, Tokyo, Japan; 3 School of Medicine, Udayana University, Bali, Indonesia

**Keywords:** Quality of life, Health utilities index Mark3 (HUI3), Japan, Translation, Validity

## Abstract

**Background:**

The McMaster Health Utilities Index Mark 3 (HUI3) is a generic multi-attribute, preference-based system for assessing health-related quality of life (HRQOL). This study describes the translation procedures and cultural adaptation of the Japanese HUI3 and its measurement properties in a community sample.

**Methods:**

The Japanese HUI3 was developed through forward and back translations in cooperation with the developers of the HUI. Acceptability, comprehensibility of questionnaires, and test-retest reliability were assessed. In a community survey of a total of 3860 people (age: 41 ± 14.3, male/female: 2651/1209), the Canadian scoring function was used to calculate utility scores. Construct validity was assessed by examining the relationship between 20 personal characteristics and utility scores.

**Results:**

Linear regression estimates demonstrated a significant negative relation between HUI3 utility score and low education, male gender, poor interpersonal relationships, older age, and a higher number of chronic diseases. Single-attribute utility scores were associated with chronic conditions in the manner expected. The community samples were relatively healthy. More than 90% of the respondents were distributed in levels 1 and 2 in all attributes except cognition. Interpretability of utility score was assessed by estimation of the relationship between visual analogue scale (VAS) and the self-rated health and utility score. Independence of attributes was assessed. For only 3 of the 28 possible cross-comparisons among the 8 attributes were correlations coefficients greater than 0.25.

**Conclusion:**

Translation and adaptation of the HUI3 questionnaire into Japanese was successful, but the sample size and selection bias limit the interpretation of our study conclusions.

## Introduction

Assessment of health-related quality of life (HRQOL) is an essential element of health care evaluations and is performed using specialized measuring tools [1]. Some of these tools can be categorized as generic HRQOL instruments, meaning that they are designed to be applicable across a wide range of populations and interventions. One such generic HRQOL instrument is the Health Utilities Index Mark 3 (HUI3). The HUI3 provides a comprehensive framework within which to measure health status and calculate HRQOL scores that can be used in economic evaluations, such as analysis of cost per quality-adjusted life year (QALY). The HUI3 is comprised of two complementary components. The first component is a multi-attribute health status classification system that is used to describe health status, and the second is a multi-attribute utility function that is used to evaluate health status assessed through the multi-attribute health-classification system of the previous component. The system defines 972,000 unique health statuses, as it focuses on eight attributes (vision, hearing, speech, ambulation, dexterity, emotion, cognition, and pain or discomfort), with each stratified into 5–6 functional levels. The minimum important difference (MID) for the HUI3 has been estimated to be 0.03 [2, 3], and 0.01 for population health applications [3]. A multi-attribute preference function for the HUI3 has been developed in Canada (details regarding this are presented elsewhere [4–7]); furthermore, in addition to providing utility scores determined to be reflective of Canadian community health preferences, the HUI3 was the sole utility score measurement instrument administered to respondents of the 2013–2014 Canadian Community Health Survey [8, 9].

The HUI3 system has been implemented in four large-scale Canadian population health surveys: the 1990 Ontario Health Survey [10, 11], the 1991 General Social Survey [12], the National Population Health Surveys [13, 14], and the National Longitudinal Surveys of Children and Youth [15]. Supporting its use is the fact that HUI measures have been proven to be reliable [16, 17] and to capture the pertinent attributes of health status for the general population [18, 19]. Recently, in a large Canadian community survey (the actual sample contained 128,310 individuals, which was adjusted by weighting to correspond to 30,014,589 individuals), Guertin et al. reported age- and sex-specific HUI3 utility score norms that enabled them to perform adequate inter-group comparisons [20].

In Japan, several generic instruments have been translated for use regarding general population samples. Fukuhara et al. conducted a translation, adaptation, and validation study of the SF-36 Health Survey [21]. Further, the Japanese EuroQol (EQ-5D-3 L) Development Committee reported on the official Japanese version of EuroQol [22], and Ikeda et al. used the Japanese EuroQol instrument to determine the health status of Japanese populations [23]. Moreover, Tsuchiya et al. estimated an EQ-5D-3 L population value set for Japan [24] and Ikeda et al. developed a Japanese version of the EuroQol 5-dimension-5-level (EQ-5D-5 L) value set [25]. Finally, Tazaki et al. conducted a qualitative and field study of cancer patients using the WHOQOL instrument [26], which they had translated into Japanese following the strict protocol required by the original developers.

In order to conduct international comparison of health status using HRQOL-measurement instruments, translations of such instruments are necessary that ensure that the meanings of the translated items are as close as possible to those of the original items. To appropriately perform such translation, a process involving three major steps must be applied: 1) forward translation, 2) backward translation and review by the original developers, and 3) testing focus groups. Considering the Japanese context, as Japanese is not part of the Indo-European language family and as Western culture is not dominant in Japan, conceptual difficulties when translating certain words from Western languages to Japanese or vice-versa are not uncommon; this is significant, as the overall goal is to produce a conceptual rather than literal translation that is compatible with the original meaning of the questionnaire.

For this study, in which we seek to create a Japanese version of the HUI3, we followed several published protocols regarding translation and worked closely with the original developers of the HUI. Recently, using Japanese version questionnaires, Shiroiwa et al. reported Japanese population norms for three preference-based measures: EQ-5D-3 L, EQ-5D-5 L, and SF-6D [27]; this provided useful information on conducting economic evaluations in Japan using QALYs and for mapping projects involving multi-attribute utility instruments (MAUI). However, as its 972,000 unique health status values entails higher sensitivity (for example, the EQ-5D-3 L defines 245 health status, which frequently raises the issue of ceiling effects), a Japanese version of the HUI3 could make a significant contribution to research. This article chronologically describes the process of the translation, cultural adaptation, and testing using a focus group of the Japanese version of the HUI3. Further, the results of a community survey conducted to obtain evidence of the validity of the translated and adapted HUI3-based instrument is described, along with its test-retest reliability, conceptual validity, interpretability, and construct validity.

## Material and methods

### Translation and cultural adaptation

#### Forward translation and the reconciliation version

To begin the process, the original English-language questionnaire of the HUI3 was obtained from the HUI developers. The questionnaire acquires information from respondents for classification; the health status classification system for the HUI3 is summarized in Table 1. Translators 1 and 2, who were bilingual in Japanese and English (native speakers of Japanese) independently translated the instruction items and the questionnaire from English to Japanese, thereby producing two initial Japanese versions. Both translators and the development team for the Japanese HUI then discussed the translation and conceptual problems of the two versions, consequently producing a single reconciled version.

**Table 1:** HUI Mark III health status classification system

Attribute	Level	Level description
Vision	1	Able to see well enough to read ordinary newsprint and recognize a friend on the other side of the street, without glasses or contact lenses
	2	Able to see well enough to read ordinary newsprint and recognize a friend on the other side of the street, but with glasses
	3	Able to read ordinary newsprint with or without glasses but unable to recognize a friend on the other side of the street, even with glasses
	4	Able to recognize a friend on the other side of the street with or without glasses but unable to read ordinary newsprint, even with glasses
	5	Unable to read ordinary newsprint and unable to recognize a friend on the other side of the street, even with glasses
	6	Unable to see at all
Hearing	1	Able to hear what is said in a group conversation with at least three other people, without a hearing aid
	2	Able to hear what is said in a conversation with one other person in a quiet room without a hearing aid, but requires a hearing aid to hear what is said in a group conversation with at least three other people
	3	Able to hear what is said in a conversation with one other person in a quiet room with a hearing aid, and able to hear what is said in a group conversation with at least three other people with a hearing aid
	4	Able to hear what is said in a conversation with one other person in a quiet room without a hearing aid, but unable to hear what is said in a group conversation with at least three other people even with a hearing aid
	5	Able to hear what is said in a conversation with one other person in a quiet room with a hearing aid, but unable to hear what is said in a group conversation with at least three other people even with a hearing aid
	6	Unable to hear at all
Speech	1	Able to be understood completely when speaking with strangers or friends
	2	Able to be understood partially when speaking with strangers but able to be understood completely when speaking with people who know the respondent well
	3	Able to be understood partially when speaking with strangers or people who know the respondent well
	4	Unable to be understood when speaking with strangers but able to be understood partially by people who know the respondent well
	5	Unable to be understood when speaking to other people (or unable to speak at all)
Amublation	1	Able to walk around the neighborhood without difficulty, and without walking equipment
	2	Able to walk around the neighborhood with difficulty, but does not require walking equipment or the help of another person
	3	Able to walk around the neighborhood with walking equipment, but without the help of another person
	4	Able to walk only short distances with walking equipment, and requires a wheelchair to get around the neighborhood
	5	Unable to walk alone, even with walking equipment; able to walk short distances with the help of another person, and requires a wheelchair to get around the neighborhood
	6	Cannot walk at all
Dexterity	1	Full use of two hands and ten fingers
	2	Limitations in the use of hands or fingers, but does not require special tools or help of another person
	3	Limitations in the use of hands or fingers, is independent with use of special tools (does not require the help of another person)
	4	Limitations in the use of hands or fingers, requires the help of another person for some tasks (not independent even with use of special tools)
	5	Limitations in use of hands or fingers, requires the help of another person for most tasks (not independent even with use of special tools)
	6	Limitations in use of hands or fingers, requires the help of another person for all tasks (not independent even with use of special tools)
Emotion	1	Happy and interested in life
	2	Somewhat happy
	3	Somewhat unhappy
	4	Very unhappy
	5	So unhappy that life is not worthwhile
Cognition	1	Able to remember most things, think clearly and solve day to day problems
	2	Able to remember most things, but have a little difficulty when trying to think and solve day to day problems
	3	Somewhat forgetful, but able to think clearly and solve day to day problems
	4	Somewhat forgetful, and have a little difficulty when trying to think or solve day to day problems
	5	Very forgetful, and have great difficulty when trying to think or solve day to day problems
	6	Unable to remember anything at all, and unable to think or solve day to day problems
Pain	1	Free of pain and discomfort
	2	Mild to moderate pain that prevents no activities
	3	Moderate pain that prevents a few activities
	4	Moderate to severe pain that prevents some activities
	5	Severe pain that prevents most activities

#### Back translation and revision by developers

The reconciled version was then translated back into English by two professional translators (Translators 3 and 4), who were bilingual and bicultural in English and Japanese (native speakers of English). Again, this was conducted independently, and two back-translated versions were thus produced. The Japanese HUI development team then compared these back-translated versions with the original English questionnaire. A critical review by the developers of the original HUI was then obtained. Through discussions between the Japanese HUI development team and the HUI developers, several linguistic, cultural, and conceptual problems were identified. These problems and the measures taken to address them are described briefly in the following sections.

#### General directions

The original English version of the HUI3 investigates respondents’ “ability” regarding each attribute except emotion. For the Japanese translation, among several options, we chose the literal translation *nouryoku* in order to avoid respondents mistaking “physical ability” for “usual practice.” In other words, the focus of the questions is what the respondent’s health status permits him/her to do or inhibits him/her from doing, not what he/she chooses to do.

Additionally, HUI23SU15Q, which is a combination of questions for HUI2 and HUI3 (a total of 15 questions) where “S” and “U” represent “Self-Assessment” and “Usual,” respectively, includes several questions with similar wording, so in order to encourage respondents to think carefully about their responses for each question, in the instructions provided at the beginning of the survey we asked respondents to “please excuse any apparent overlap between questions and answer each independently.”

##### Vision

For the section relating to vision, the translated question emphasized that the items concerned the ability to see, not how well the respondent could read; in other words, the item concerns eyesight quality, not literacy. This concept was reflected in both the question and response options, with the concept of “being able to see or distinguish” emphasized in the wording of the translation.

##### Hearing

Similar to the concept of vision, the focus of the hearing item concerns hearing, not comprehension. This concept is reflected in the wording of the Japanese translation.

##### Speech

The concept present in and the best translation of “when speaking with people who know you well” were discussed with the developer. It was finally decided that “people who know you well” should indicate people who are very familiar with the respondent. “Your own language,” which was present in the item in the original questionnaire, was omitted because almost all respondents in Japan are native speakers of Japanese.

##### Ambulation

The cultural concept of “neighborhood” was difficult to translate into Japanese. Eventually, the wording of the Japanese version was set to convey to the respondents the ability to walk several hundred meters outdoors in a non-challenging environment. Further, the intent of the question is not restricted to walking but concerns physically moving about in general; the corresponding Japanese item conveys this concept.

##### Dexterity

The appropriate mean of conveying the concept of “special tools” was discussed with developer, as the tools described in the original questionnaire are not common in Japanese daily life and culture. Additionally, “limitation” was translated as “not free to do” in order to appropriately convey the concept.

##### Emotion

As is often the case, the translation and cultural adaptation of emotional concepts was difficult. The appropriate means of conveying the concept of “somewhat” was discussed with developer (e.g., “somewhat happy”); the translation was eventually set to convey a range of intensity comprising five levels. To assist in selecting the best Japanese words for this range, magnitude estimation was employed by the translator and the development team using a visual analogue scale (VAS).

##### Cognition

For cognition, the translation focused on the severity of cognitive problems (i.e., remembering or thinking), rather than the frequency of such problems.

##### Pain

For pain, the appropriate means of conveying the original concept, the frequency and severity of pain, were discussed with the developer. Cultural differences regarding means of relieving pain were also taken into account. For example, in Japan, people often cast a spell or pray to relieve pain.

### Revision and second back translation

Close attention was paid to the problems identified in the back translation and the reconciled version was corrected accordingly. Several lay panel sessions (featuring different groups) provided suggestions on means of conveying the original concepts in Japanese while maintaining natural and appropriate language. Small focus group testing was then conducted with a sample of 158 community respondents, who were asked to report any difficulties they experienced regarding the concept of the questionnaire and the response options. Consequently, very few problems were identified, and the questionnaire was considered suitable for use with a Japanese sample. All procedures were then reported to the HUI developers, together with a second back translation. The HUI developers and Japanese HUI development team were satisfied with the results and the Japanese HUI23SU15Q was then deemed ready for use in Japan.

### Japanese community survey

A large community survey was conducted in the fall of 1999. Overall, 3860 people, comprising employees of two large corporations and members of their families and nearby residents, 200 residents of the Shizuoka District (200 km west of Tokyo), were included as respondents. The HUI questionnaire was distributed to each respondent individually via local branches of the two corporations or at the time of their visit to the company clinic for a routine health checkup. Questionnaires were returned by mail to the respective branches of the corporations and were then mailed to the author’s office.

Along with the HUI questionnaire, respondents were asked to complete the VAS task. This task comprised a vertical thermometer-shaped scale nine cm long; the top was labeled “1.0,” representing *perfect health*, and the bottom was labeled “0,” *dead*; respondents were asked to imagine their usual health status and mark the point on this scale that corresponded to it. This VAS estimation is not part of the original HUI questionnaire. Respondents were also asked to answer items concerning 20 personal characteristics potentially related to HRQOL, the same variables as those surveyed in the Ontario Health Survey [10, 11], specifically: Name, sex, age, BMI (body mass index), survey date, occupation, level of education, residential area, family size, marital status, type of residence, annual family income, work schedule, employment conditions, job stability, commuting time, quality of interpersonal relationships at work over the past three months, quality of interpersonal relationships at home over the past three months, and number and type of chronic diseases.

In order to calculate multi-attribute global utility scores and single-attribute utility scores, the HUI3 scoring function designed by Furlong et al. was adopted [28]. The single-attribute and global scoring functions for the original HUI3 are based on data collected from a preference survey of a random sample of the general population of Hamilton, Ontario, Canada. The HUI3 global utility scores range from − 0.36 to 1.00 (indicating perfect health); the negative lower bound reflects the fact that in the preference survey respondents judged the health state that corresponded to the lowest level of capacity in each of the eight attributes to be worse than death [29].

### Statistical analysis

This study was designed to validate the Japanese version of the HUI3 for use in Japan, so we mainly focused on the distribution of attribute levels and mean utility scores. We also examined the relationships between personal characteristics and HUI3 and VAS scores. Furthermore, we investigated the ability of personal characteristics to predict HUI3 scores among this community sample.

### Reliability

Reliability was examined using a community sample (*n* = 112). They completed the same questionnaire after a three-week interval, and utility scores (multi-attribute global, single-attribute) and a VAS (visual analogue scale) score were analyzed for intraclass correlation coefficients (ICCs) to assess test-retest reliability among the two data sets. Focus group members who were close to the authors were asked to report any health status changes and usual condition changes, if any, over the three-week interval. Additionally, correlation coefficients were calculated to assess how differences depended on age group, personal characteristics, such as with or without chronic disease, and status of interpersonal relationships in the family and work site.

### Validity

In order to assess construct validity, the relationships between personal characteristics and HUI3 scores were examined. First, we compared the mean global utility and single-attribute utility scores in term of groups created based on the personal characteristic variables. The categories were the following. For age group: “younger than 20” (12–19), “20–29,” “30–39,” “40–49,” “50–59,” “60–69,” or “70 and older”; for level of education: “student,” “low,” or “high”; for marital status: “married,” “divorced,” “widowed,” or “single”; for gender: “male” or “female”; for annual family income: “US$0–10,000,” “US$10,000–50,000,” or “more than US$50,000” (US$1 ≒ JPY110 in 1999); for employment: “seeking work or part-time worker,” “student,” “housewife,” or “other”; for interpersonal relationship in the workplace and among the family: “excellent,” “good,” “fair,” “bad,” or “very bad”; and for number of chronic diseases: “0,” “1,” “2,” or “3.”

Respondents were assigned to the high-education category if they had at least a college degree, while those with elementary school, junior high school, high school, vocational school, or other levels of education were assigned to the low education category. Family income was included in the respondents’ annual household income. If the respondents were students, any income obtained through part-time work and from parents was included; if the respondents were housewives, their husband’s income served to indicate their income. For employment, respondents were allocated to the categories “employed/ seeking work,” “part-time job/retired,” or “unemployed.” The number of chronic diseases reflected the number of chronic diseases the respondents had; they answered this item by checking as many of the following options as applicable: “allergies,” “asthma,” “arthritis,” “back pain or other back problems,” “high blood pressure,” “migraine headaches,” “chronic bronchitis or emphysema,” “sinusitis,” “diabetes,” “epilepsy,” “heart disease,” “cancer,” “stomach or intestinal ulcers,” “effects of stroke,” “urinary incontinence,” “liver dysfunction,” “dermatitis requiring medication,” “dementia,” “cataracts,” or “other chronic condition.” This list of chronic diseases was sourced from the Ontario Health Survey [10, 11]. If our translation is appropriate and the Japanese HUI3 system valid, then lower HRQOL should on average be reflected in a lower mean global utility score and lower single-attribute utility scores.

To clarify the relationship between HUI attributes and each disease- or condition-specific problem, the 20 types of chronic disease were classified into the following 10 categories of chronic conditions determined by cardiopulmonary, neurology, and orthopedic surgery specialists in the authors’ group mainly based on the disease-specific nature of subjective symptoms as follows: “allergy,” “cardiopulmonary disease,” “musculoskeletal disorder,” “hypertension,” “hyper-lipidemia,” “metabolic disease,” “visual and hearing disorder,” “central nervous disorder,” “malignant tumor,” “gastrointestinal disorder,” and “no chronic disease.” Mean single-attribute scores, global utility scores, and VAS scores were assessed for each of these 10 categories. If the Japanese HUI3 system is valid, single utility scores should predict certain disease-specific problems; for example, respondents with central nervous disorders should report lower mean cognition utility scores.

To clarify the relationships between personal characteristic variables and HUI3 scores, linear regression models were used to compare utility scores between groups while controlling for the effects of potentially confounding variables; failure to control for confounding effects would lead to biased results. For example, mean age was related to the number of chronic diseases, widowed status, and lower education; thus, differences in health status between these groups would likely be due to the effects of both age- and personal characteristic-related variables, rather than the effects of each personal characteristic alone, as would be implied by uncontrolled comparisons of utility scores. Categorical variables were captured as dummy variables for multiple regression analysis. For statistical analysis, IBM SPSS Statistics 24 was used.

The set of control variables included respondents’ education levels, marital status, gender, annual family income, employment, interpersonal relationships in the workplace, interpersonal relationships in the family, age, and number of chronic diseases. Questions on interpersonal relationships in the family and workplace were simple multiple-choice responses scored on five levels (from *very bad* to *excellent*), which were used in the QOWL (Quality of Working Life) survey reported at the annual meeting of the Japanese Society of Hygiene in 1998 [30]. The groups and categories were the same as those described above regarding in the assessment of mean utility scores, except that age was included as a continuous variable. After excluding respondents who did not answer the respective questions, the responses of 2960 subjects were eligible for model estimation. Linear regression estimates were also conducted for the models to obtain the multi-attribute global utility score, single-attribute utility scores, and VAS scores as functions of age and of the 10 categories of chronic condition; the baseline category represented respondents who did not have any type of chronic disease (no chronic condition). After removing incomplete respondents and analyzing respondents with more than two chronic diseases independently on each chronic disease name, the number of sample participants used to estimate the regression models was 3762 for the single-attribute and global utility scores and 3576 for the VAS score, respectively. If the Japanese HUI3 has reasonable construct validity, respondents in groups with lower HRQOL-related personal characteristics should return a negative correlation coefficient between global utility score and variable categories. Furthermore, in the regression model concerning the relationship between chronic conditions and utility scores, respondents with a specific chronic condition should show a negative regression coefficient between the single-attribute utility score and the category associated with the disease-specific problem for the condition in question, such as cognition and central nervous disorder, or pain and musculoskeletal disorder, respectively.

Kendall correlations between the HUI3 single-attribute scores were calculated in order to estimate the independence of each of the eight attributes of the Japanese HUI3. If each attribute was independent, no substantial linear correlations would be found among the 28 possible cross-comparisons. The relationship between multi-attribute global HUI3 utility score and self-rated health was also estimated using the response to the self-rated health question: “Overall, how would you rate your usual health?” The possible responses were *excellent*, *very good*, *good*, *fair*, and *poor*. Additionally, we estimated the distribution (percentage) of three categories of self-rated health (*excellent or very good*, *good*, *fair or poor*) among the 10 groups of respondents with the following global utility scores: less than 0.2, 0.2 to less than 0.3, 0.3 to less than 0.4, 0.4 to less than 0.5, 0.5 to less than 0.6, 0.6 to less than 0.7, 0.7 to less than 0.8, 0.8 to less than 0.9, 0.9 to less than 1.0, and 1.0. The relationship between multi-attribute global utility scores and VAS scores was also examined. These two approaches contributed to determining whether the Japanese HUI3 material correlates with subjective (self-rated) health status.

## Results

For test-retest reliability (*n* = 104), ICCs for the global utility score was 0.84 and was 0.78, 0.93, 0.73, 0.96, 0.80, 0.44, 0.62, and 0.73 for the single-attribute scores for vision, hearing, speech, ambulation, dexterity, emotion, cognition, and pain, respectively, while for the VAS score, it was 0.79.

For the larger community survey, featuring 3860 subjects, the mean age was 41 ± 14.3 years; age ranged from 14 to 90 years, with a median of 39 and a mode of 37. The male-to-female ratio was 2651:1209; thus, there were twice as many male as female respondents. The age distribution by 10-year groups (from 10s to 70 and over) was as follows: 3.6, 17.6, 29.7, 23.2, 15.5, 5.7, and 4.7%, respectively. Sixty percent of those surveyed lived in the greater Tokyo Metropolitan Area, while the remaining 40% were distributed throughout the nation. For the survey, the respondent and administrative burden was determined to be acceptable.

Distribution of single-attribute levels among the respondents is shown in Table 2. No respondent had level 6 hearing, speech, dexterity, emotion, or pain. Meanwhile, approximately 100% had level 1 hearing, speech, ambulation, and dexterity, and the distributions of the respondents with level 1 and level 2 vision, emotion, and pain were almost identical. Table 3 presents the means and standard deviations for the utility scores (single-attribute and multi-attribute global) and VAS scores of the 10-year age groups. For all attributes, there was no age-related decline for age groups younger than 70 years. ANOVA revealed significant differences in mean utility score (single-attribute and multi-attribute global) and VAS score for the over-70 group, showing that age-related decline begins at this age. For emotion, significantly lower utility scores were seen in younger groups. Furthermore, the global utility score was substantially lower for the 40s and 50s age groups, and VAS score declined as age increased.

**Table 2:** Distribution of single attributes level by all age-group(%)

Level /Attributes	Vision *n* = 3752	Hearing *n* = 3752	Speech *n* = 3752	Ambulation *n* = 3752	Dexterity *n* = 3752	Emotion *n* = 3752	Cognion *n* = 3752	Pain *n* = 3752
Level 1	39.13	91.71	91.71	98.56	98.72	43.63	49.33	44.08
Level 2	58.8	4.1	4.1	0.85	1.04	49.39	4.96	47.68
Level 3	1.28	3.97	3.97	0.45	0.19	6.1	28.73	6.53
Level 4	0.43	0.16	0.16	0.05	0	0.72	14.61	1.39
Level 5	0.35	0.05	0.05	0.05	0.05	0.16	2.29	0.32
Level 6	0.03	0	N.A.	0.03	0	N.A.	0.08	N.A.

N.A. Not applicable; There is no level 6 for that attribute

**Table 3:** Mean Utility Scores (Single Attribute, Multiattribute Global HUI3)and VAS score by Age Group

age-group	n	single attributes									
		Vision^**^	Hearing^**^	Speech^**^	Ambulation^**^	Dexterity^**^	Emotion^**^	Cognition^**^	Pain^**^	Global^**^	VAS^**^
0–19	136	0.97 ± 0.08	1.00 ± 0.00	0.96 ± 0.12	1.00 ± 0.01	1.00 ± 0.01	0.92 ± 0.13	0.88 ± 0.19	0.93 ± 0.14	0.85 ± 0.18	0.82 ± 0.19
20–29	661	0.97 ± 0.05	1.00 ± 0.00	0.98 ± 0.08	1.00 ± 0.01	1.00 ± 0.01	0.93 ± 0.09	0.93 ± 0.13	0.94 ± 0.09	0.87 ± 0.13	0.86 ± 0.14
30–39	1113	0.97 ± 0.05	1.00 ± 0.04	0.99 ± 0.06	1.00 ± 0.03	1.00 ± 0.03	0.93 ± 0.09	0.92 ± 0.13	0.94 ± 0.09	0.87 ± 0.13	0.85 ± 0.13
40–49	871	0.97 ± 0.06	1.00 ± 0.05	0.97 ± 0.08	1.00 ± 0.02	1.00 ± 0.01	0.93 ± 0.10	0.90 ± 0.14	0.94 ± 0.09	0.83 ± 0.16	0.82 ± 0.15
50–59	583	0.95 ± 0.06	0.99 ± 0.08	0.97 ± 0.10	1.00 ± 0.01	1.00 ± 0.01	0.93 ± 0.09	0.89 ± 0.14	0.94 ± 0.07	0.82 ± 0.16	0.81 ± 0.14
60–69	212	0.95 ± 0.06	0.99 ± 0.07	0.99 ± 0.05	1.00 ± 0.03	1.00 ± 0.02	0.96 ± 0.05	0.91 ± 0.11	0.93 ± 0.08	0.87 ± 0.11	0.79 ± 0.17
70 and over	176	0.94 ± 0.09	0.93 ± 0.20	0.97 ± 0.09	0.94 ± 0.14	0.98 ± 0.08	0.95 ± 0.07	0.85 ± 0.19	0.87 ± 0.19	0.74 ± 0.24	0.67 ± 0.22
all age	3752	0.96 ± 0.06	0.99 ± 0.07	0.98 ± 0.08	1.00 ± 0.04	1.00 ± 0.02	0.93 ± 0.09	0.91 ± 0.14	0.94 ± 0.10	0.85 ± 0.15	0.83 ± 0.15

Values represent the arithmetic means±SD *; *p* < 0.05, **; *p* < 0.01 by ANOVA (among age-group)

Tables 4 and 5 shows the means and standard deviations of the utility and VAS scores for each personal characteristic-based group. For the single-attribute utility score, several substantially lower scores were observed. For instance, for hearing, ambulation, cognition, and pain, individuals who were widowed and who were seeking work or were working part-time showed lower single-attribute utility scores. Moreover, for cognition, lower levels of educational attainment showed lower scores. Regarding interpersonal relationships in the workplace and the family and the number of chronic conditions, single-attribute and global utility scores showed rank correlations; for example, worse categories of inter-human relationship ware associated with lower scores in both single-attribute and global utility. Finally, lower global utility scores were observed for low education, widowed, male gender, higher annual family income, and seeking work or working part-time.

**Table 4:** Mean utility scores (single attribute, multiattribute global HUI3) and VAS score by personal characteristic variables

Variables and category	n	age	Attributes and single utility scores			
		avg ± std	Vision	Hearing	Speech	Ambulation
Education						
Student	194	19.88 ± 3.07	0.96 ± 0.08	1.00 ± 0.00	0.98 ± 0.07	1.00 ± 0.00
Low	1515	46.06 ± 12.87	0.96 ± 0.06	0.99 ± 0.08	0.97 ± 0.10	1.00 ± 0.05
High	1743	37.67 ± 10.84	0.96 ± 0.05	1.00 ± 0.05	0.99 ± 0.06	1.00 ± 0.03
Marital status						
Married	2413	44.63 ± 11.54	0.96 ± 0.06	0.99 ± 0.06	0.98 ± 0.08	1.00 ± 0.03
Divorced	131	42.84 ± 10.93	0.97 ± 0.04	1.00 ± 0.02	0.99 ± 0.05	0.99 ± 0.06
Widowed	103	70.50 ± 11.79	0.94 ± 0.08	0.94 ± 0.19	0.96 ± 0.10	0.95 ± 0.12
Single	894	27.77 ± 8.89	0.97 ± 0.06	1.00 ± 0.03	0.97 ± 0.09	1.00 ± 0.04
Gender						
Male	2540	40.61 ± 12.12	0.96 ± 0.06	0.99 ± 0.07	0.97 ± 0.08	1.00 ± 0.04
Female	1030	42.03 ± 18.38	0.96 ± 0.06	0.99 ± 0.07	0.99 ± 0.07	0.99 ± 0.05
Annual family income						
0-10000USD	442	37.90 ± 19.73	0.96 ± 0.06	1.00 ± 0.04	0.98 ± 0.09	0.99 ± 0.06
10,000-50000USD	1182	37.50 ± 15.38	0.96 ± 0.06	0.99 ± 0.07	0.98 ± 0.08	1.00 ± 0.04
More than 50000USD	1763	43.38 ± 8.73	0.96 ± 0.05	0.99 ± 0.06	0.98 ± 0.08	1.00 ± 0.02
Employment						
Seeking work or part time	149	69.13 ± 14.55	0.94 ± 0.11	0.94 ± 0.20	0.97 ± 0.10	0.94 ± 0.16
Student	217	20.08 ± 3.76	0.96 ± 0.07	1.00 ± 0.00	0.97 ± 0.09	1.00 ± 0.00
House wife	376	49.56 ± 14.96	0.96 ± 0.05	0.99 ± 0.04	0.99 ± 0.05	1.00 ± 0.02
Others	2800	40.01 ± 10.79	0.96 ± 0.05	1.00 ± 0.05	0.98 ± 0.08	1.00 ± 0.02
Inter-human relationship in work site						
Excellent	445	37.30 ± 12.44	0.97 ± 0.06	1.00 ± 0.03	0.99 ± 0.07	1.00 ± 0.02
Good	1230	38.90 ± 11.36	0.97 ± 0.05	1.00 ± 0.04	0.98 ± 0.06	1.00 ± 0.01
Fair	1213	40.25 ± 11.30	0.96 ± 0.06	1.00 ± 0.06	0.97 ± 0.09	1.00 ± 0.02
Bad	133	38.73 ± 9.70	0.97 ± 0.05	0.98 ± 0.09	0.95 ± 0.11	1.00 ± 0.02
Very bad	30	39.26 ± 9.15	0.98 ± 0.03	0.96 ± 0.19	0.93 ± 0.16	1.00 ± 0.00
Inter-human relationship in family						
Excellent	883	39.84 ± 14.06	0.97 ± 0.05	1.00 ± 0.03	0.99 ± 0.06	1.00 ± 0.05
Good	1446	40.83 ± 13.30	0.96 ± 0.06	0.99 ± 0.06	0.98 ± 0.07	1.00 ± 0.03
Fair	1056	42.43 ± 14.60	0.96 ± 0.06	0.99 ± 0.07	0.97 ± 0.10	1.00 ± 0.04
Bad	85	38.45 ± 13.11	0.96 ± 0.08	0.96 ± 0.19	0.96 ± 0.11	0.99 ± 0.07
Very bad	18	36.83 ± 10.42	0.98 ± 0.03	0.94 ± 0.24	0.94 ± 0.14	1.00 ± 0.00
Number of chronic disease						
3 and over	78	58.91 ± 16.79	0.94 ± 0.11	0.93 ± 0.21	0.95 ± 0.12	0.97 ± 0.08
2	222	52.41 ± 17.48	0.95 ± 0.08	0.98 ± 0.13	0.97 ± 0.09	0.98 ± 0.08
1	877	44.36 ± 15.19	0.96 ± 0.07	0.99 ± 0.06	0.97 ± 0.09	0.99 ± 0.05
0	2400	38.30 ± 12.25	0.97 ± 0.05	1.00 ± 0.04	0.98 ± 0.07	1.00 ± 0.03

value represent the arthmetic means ±SD, USD was reported as 1999 USD value converted from JPY

**Table 5:** Mean utility scores (single attribute, multiattribute global HUI3) and VAS score by personal characteristic variables

Variables and category	Dexterity	Emotion	Cognition	Pain	Global	VAS
Education						
Student	1.00 ± 0.01	0.93 ± 0.11	0.92 ± 0.14	0.95 ± 0.11	0.89 ± 0.13	0.85 ± 0.17
Low	1.00 ± 0.03	0.92 ± 0.10	0.89 ± 0.15	0.93 ± 0.11	0.81 ± 0.18	0.81 ± 0.16
High	1.00 ± 0.02	0.94 ± 0.08	0.93 ± 0.13	0.94 ± 0.08	0.88 ± 0.12	0.85 ± 0.14
Marital status						
Married	1.00 ± 0.02	0.94 ± 0.09	0.91 ± 0.13	0.94 ± 0.09	0.85 ± 0.15	0.83 ± 0.15
Divorced	1.00 ± 0.01	0.92 ± 0.10	0.90 ± 0.13	0.94 ± 0.10	0.85 ± 0.14	0.81 ± 0.17
Widowed	0.98 ± 0.06	0.94 ± 0.08	0.84 ± 0.20	0.90 ± 0.16	0.76 ± 0.23	0.68 ± 0.21
Single	1.00 ± 0.03	0.91 ± 0.11	0.91 ± 0.15	0.94 ± 0.11	0.85 ± 0.15	0.84 ± 0.15
Gender						
Male	1.00 ± 0.03	0.93 ± 0.10	0.91 ± 0.14	0.94 ± 0.10	0.84 ± 0.16	0.82 ± 0.15
Female	1.00 ± 0.02	0.94 ± 0.08	0.92 ± 0.13	0.93 ± 0.09	0.87 ± 0.13	0.83 ± 0.16
Annual family income						
0-10000USD	0.99 ± 0.04	0.94 ± 0.10	0.92 ± 0.14	0.93 ± 0.12	0.87 ± 0.16	0.83 ± 0.17
10,000-50000USD	1.00 ± 0.03	0.92 ± 0.10	0.91 ± 0.14	0.93 ± 0.10	0.84 ± 0.16	0.83 ± 0.16
More than 50000USD	1.00 ± 0.01	0.93 ± 0.09	0.91 ± 0.13	0.94 ± 0.09	0.84 ± 0.15	0.82 ± 0.15
Employment						
Seeking work or part time	0.97 ± 0.10	0.95 ± 0.08	0.85 ± 0.20	0.89 ± 0.18	0.75 ± 0.25	0.67 ± 0.23
Student	1.00 ± 0.01	0.93 ± 0.11	0.92 ± 0.15	0.94 ± 0.13	0.88 ± 0.14	0.85 ± 0.17
House wife	1.00 ± 0.02	0.96 ± 0.07	0.93 ± 0.11	0.94 ± 0.08	0.90 ± 0.10	0.84 ± 0.14
Others	1.00 ± 0.01	0.93 ± 0.10	0.91 ± 0.13	0.94 ± 0.09	0.84 ± 0.15	0.83 ± 0.14
Inter-human relationship in work site						
Excellent	1.00 ± 0.01	0.96 ± 0.09	0.94 ± 0.11	0.95 ± 0.08	0.91 ± 0.12	0.87 ± 0.14
Good	1.00 ± 0.01	0.94 ± 0.08	0.93 ± 0.12	0.95 ± 0.08	0.87 ± 0.12	0.85 ± 0.13
Fair	1.00 ± 0.01	0.91 ± 0.09	0.89 ± 0.15	0.93 ± 0.09	0.82 ± 0.15	0.81 ± 0.15
Bad	1.00 ± 0.02	0.85 ± 0.16	0.84 ± 0.19	0.88 ± 0.14	0.72 ± 0.22	0.76 ± 0.17
Very bad	1.00 ± 0.02	0.83 ± 0.21	0.79 ± 0.19	0.87 ± 0.21	0.69 ± 0.25	0.78 ± 0.22
Inter-human relationship in family						
Excellent	1.00 ± 0.03	0.96 ± 0.09	0.93 ± 0.13	0.95 ± 0.07	0.90 ± 0.13	0.87 ± 0.14
Good	1.00 ± 0.02	0.94 ± 0.07	0.92 ± 0.13	0.94 ± 0.08	0.85 ± 0.14	0.83 ± 0.15
Fair	1.00 ± 0.02	0.90 ± 0.10	0.89 ± 0.15	0.92 ± 0.12	0.80 ± 0.17	0.79 ± 0.16
Bad	0.99 ± 0.03	0.85 ± 0.17	0.84 ± 0.19	0.89 ± 0.19	0.74 ± 0.24	0.78 ± 0.20
Very bad	1.00 ± 0.00	0.77 ± 0.30	0.83 ± 0.27	0.88 ± 0.23	0.66 ± 0.30	0.72 ± 0.21
Number of chronic disease						
3 and over	0.99 ± 0.04	0.92 ± 0.13	0.83 ± 0.17	0.86 ± 0.14	0.72 ± 0.22	0.64 ± 0.23
2	0.99 ± 0.03	0.92 ± 0.12	0.88 ± 0.16	0.88 ± 0.15	0.77 ± 0.19	0.72 ± 0.19
1	1.00 ± 0.03	0.93 ± 0.10	0.90 ± 0.14	0.92 ± 0.12	0.82 ± 0.17	0.79 ± 0.16
0	1.00 ± 0.02	0.93 ± 0.09	0.92 ± 0.13	0.95 ± 0.07	0.87 ± 0.13	0.85 ± 0.13

value represent the arthmetic means ±SD, USD was reported as 1999 USD value converted from JPY

In Table 6, baseline variables (omitted category) are noted for each variable. Significant negative correlation coefficients were observed for lower educational attainment, male gender, and higher number of chronic diseases; meanwhile, significant positive correlations were observed for fair, good, and excellent (omitting “very bad”) interpersonal relationships in the family and the workplace. The intercept was 0.67 and the coefficient of determination was 0.19.

**Table 6:** Linear regression estimates for model of multiattribute global HUI3 utility score as a function of personal characteristic variables

Variables and category	coefficient	*P* Value	95% confidence Intervals	
Education				
Student	0.048	0.131	−0.014	0.110
Low	−0.054	0.000	−0.066	−0.043
High (baseline)				
Marital status				
Married	0.021	0.007	0.006	0.036
Divorced	0.025	0.079	−0.003	0.053
Widowed	−0.009	0.731	− 0.061	0.043
Single (baseline)				
Gender				
Male	−0.040	0.000	−0.055	−0.025
Female (baseline)				
Annual family income				
0-10000USD	−0.003	0.836	−0.036	0.029
10,000-50000USD	−0.012	0.061	−0.025	0.001
More than 50000USD (baseline)				
Employment				
Seeking work or part time	0.068	0.054	−0.001	0.137
Student	−0.044	0.160	−0.106	0.017
House wife	0.025	0.188	−0.012	0.062
Others (baseline)				
Inter-human relationship in work site				
Excellent	0.162	0.000	0.110	0.214
Good	0.152	0.000	0.102	0.203
Fair	0.120	0.000	0.070	0.171
Bad	0.030	0.277	−0.024	0.085
Very bad (baseline)				
Inter-human relationship in family				
Excellent	0.146	0.000	0.080	0.212
Good	0.118	0.000	0.052	0.183
Fair	0.095	0.005	0.029	0.160
Bad	0.027	0.471	−0.046	0.099
Very bad (baseline)				
Age	−4.57E-04	1.63E-01	−1.10E-03	1.85E-04
Number of chronic disease				
three and more	−0.057	0.022	−0.106	− 0.008
two	−0.072	0.000	−0.096	− 0.047
one	−0.025	0.000	−0.037	− 0.012
none (baseline)				
Constant	0.667	0.000	0.584	0.750
R2				0.192

USD was reported as 1999 USD value converted from JPY

We also examined the mean utility and VAS scores for other personal characteristic variables, such as BMI (height and weight), occupation, residential area, family size, type of residence, debt, work schedule, job stability, and commuting time. This did not reveal any systematic associations. Reasons for this may include the respondents misunderstanding some questions, such as that regarding type of residence; several respondents reported their ownership status, but the question actually concerned space and comfort. Another possible reason is that the sample size was small.

Table 7 provides the mean single-attribute and global utility scores and VAS scores for each type of chronic disease. Respondents with any type of chronic disease returned comparatively lower single-attribute utility scores for all attributes. Hyper-lipidemia corresponded to lower single-attribute utility scores for vision, as did malignant tumor for hearing and allergy, metabolic disease for speech, visual and hearing disorder for ambulation and cardiopulmonary disease, musculoskeletal disorder and central nerve disorder for emotion, central nervous disorder for cognition, and musculoskeletal disorder for pain. Regarding global utility and VAS scores, the highest mean scores were found for groups with no chronic disease. Table 8 shows the results of a linear regression model for global, single-attribute, and VAS scores as a function of age and type of chronic disease; the baseline category represents respondents with no chronic disease. With respect to single-attribute utility scores, the following significantly negative correlations were found: Between allergy and speech, emotion, cognition, and pain; between cardiopulmonary disease and hearing, speech, emotion and pain; between musculoskeletal disorder and dexterity and pain; between hyper-lipidemia and emotion; between metabolic disease and speech; between visual and hearing disorder and ambulation and pain; between central nervous disorder and vision, hearing, ambulation, dexterity, cognition, and pain; between malignant tumor and vision, hearing, ambulation, dexterity, and pain; and between gastrointestinal disorder and pain. Age was used in regression estimates as a continuous variable and consequently showed a significant negative correlation with global utility score, VAS score, and all single-attribute utility scores except speech. With respect to global utility score, significant negative correlations were observed for allergy, cardiopulmonary disease, musculoskeletal disorder, metabolic disease, visual and hearing disorder, central nerve disorder, and gastrointestinal disorder. The number of subjects used in these regression estimates was 3762 for single-attribute and global utility scores and 3576 for VAS score.

**Table 7:** Mean Utility Scores (Single Attribute, Multiattribute Global HUI3)and VAS score by Type of Chronic disease

Chronic disease	n	age	attributes									
		avg ± std	Vision	Hearing	Speech	Ambulation	Dexterity	Emotion	Cognition	Pain	Global	VAS
No chronic disease	2570	38.30 ± 12.25	0.97 ± 0.05	1.00 ± 0.04	0.98 ± 0.07	1.00 ± 0.03	1.00 ± 0.02	0.93 ± 0.09	0.92 ± 0.13	0.95 ± 0.07	0.87 ± 0.13	0.85 ± 0.13
Allergy	152	41.77 ± 12.76	0.97 ± 0.05	0.98 ± 0.10	0.95 ± 0.11	0.99 ± 0.04	1.00 ± 0.01	0.92 ± 0.09	0.88 ± 0.16	0.91 ± 0.11	0.76 ± 0.19	0.76 ± 0.17
Cardiopulmonary disease	187	47.50 ± 17.22	0.96 ± 0.07	0.98 ± 0.13	0.96 ± 0.10	0.99 ± 0.03	1.00 ± 0.02	0.91 ± 0.14	0.89 ± 0.16	0.90 ± 0.14	0.79 ± 0.20	0.73 ± 0.19
Musculo-skeletal disorder	131	49.34 ± 15.05	0.95 ± 0.07	0.98 ± 0.10	0.97 ± 0.08	0.98 ± 0.07	0.99 ± 0.03	0.92 ± 0.11	0.86 ± 0.15	0.83 ± 0.16	0.73 ± 0.19	0.74 ± 0.20
Hypertension	301	58.76 ± 13.67	0.95 ± 0.08	0.98 ± 0.13	0.97 ± 0.10	0.98 ± 0.08	0.99 ± 0.05	0.94 ± 0.08	0.89 ± 0.13	0.92 ± 0.12	0.80 ± 0.18	0.73 ± 0.19
Hyper Lipidemia	19	56.79 ± 13.60	0.93 ± 0.13	1.00 ± 0.00	0.99 ± 0.04	0.98 ± 0.07	0.99 ± 0.06	0.98 ± 0.04	0.94 ± 0.11	0.90 ± 0.15	0.83 ± 0.16	0.81 ± 0.13
Metabolic disease	117	55.45 ± 13.30	0.95 ± 0.08	0.98 ± 0.10	0.95 ± 0.12	0.99 ± 0.05	1.00 ± 0.02	0.94 ± 0.07	0.87 ± 0.16	0.92 ± 0.10	0.78 ± 0.20	0.71 ± 0.21
Visual & hearing disorder	71	68.17 ± 13.20	0.94 ± 0.09	0.96 ± 0.16	0.97 ± 0.09	0.96 ± 0.11	0.99 ± 0.03	0.95 ± 0.05	0.86 ± 0.16	0.89 ± 0.16	0.75 ± 0.21	0.67 ± 0.23
Central nervous disorder	111	43.91 ± 17.89	0.94 ± 0.10	0.97 ± 0.16	0.96 ± 0.11	0.97 ± 0.12	0.99 ± 0.05	0.91 ± 0.10	0.84 ± 0.19	0.85 ± 0.17	0.74 ± 0.21	0.75 ± 0.21
malignant tumor	22	59.82 ± 13.09	0.93 ± 0.12	0.95 ± 0.21	0.97 ± 0.09	0.97 ± 0.13	0.99 ± 0.06	0.95 ± 0.06	0.92 ± 0.16	0.87 ± 0.22	0.82 ± 0.24	0.70 ± 0.26
Gastro intestinal disorder	179	49.52 ± 13.96	0.95 ± 0.08	0.97 ± 0.14	0.97 ± 0.09	0.99 ± 0.06	0.99 ± 0.03	0.93 ± 0.08	0.89 ± 0.14	0.91 ± 0.13	0.80 ± 0.18	0.74 ± 0.18

Values represent the arithmetic means±standard deviations

**Table 8:** Linear regression estimates for model of utility score (single attribute, multiattribute global HUI3) and VAS score as a function of age and type of chronic disease

Chronic disease	attributes *n* = 3762								global *n* = 3762	VAS *n* = 3576
	Vision	Hearing	Speech	Ambulation	Dexterity	Emotion	Cognition	Pain		
(constant)	0.983	1.023	0.983	1.014	1.005	0.918	0.952	0.961	0.912	0.911
	0.000	0.000	0.000	0.000	0.000	0.000	0.000	0.000	0.000	0.000
age	−4.39E-04	−6.63E-04	−2.67E-05	−4.14E-04	−1.69E-04	4.29E-04	−8.82E-04	−3.69E-04	−1.25E-03	−1.58E-03
	0.000	0.000	0.794	0.000	0.000	0.000	0.000	0.003	0.000	0.000
Allergy	−0.007	−0.005	−0.013	6.06E-05	0.002	−0.011	− 0.021	− 0.021	− 0.032	− 0.028
	0.018	0.129	0.002	0.976	0.204	0.026	0.004	0.000	0.000	0.000
Cardiopulmonary disease	1.05E-04	−0.012	− 0.014	2.08E-04	3.66E-04	−0.024	− 0.010	− 0.027	− 0.040	−0.068
	0.981	0.015	0.020	0.944	0.847	0.001	0.326	0.000	0.001	0.000
Musculo-skeletal disorder	−0.002	0.002	− 0.004	−0.003	− 0.008	−0.011	− 0.028	−0.097	− 0.079	−0.063
	0.740	0.749	0.621	0.462	0.007	0.309	0.078	0.000	0.000	0.000
Hypertension	−0.004	−0.002	−0.003	− 0.003	−0.001	− 0.002	−1.54E-04	−0.008	− 0.016	−0.053
	0.247	0.638	0.520	0.273	0.362	0.685	0.986	0.181	0.110	0.000
Hyper Lipidemia	−0.020	0.021	0.015	−0.003	− 0.008	0.044	0.045	−0.025	0.019	0.029
	0.132	0.170	0.413	0.718	0.147	0.042	0.156	0.253	0.577	0.394
Metabolic disease	−0.005	0.005	−0.027	0.002	0.003	0.002	−0.027	−0.010	− 0.045	−0.075
	0.441	0.449	0.001	0.683	0.255	0.835	0.059	0.318	0.003	0.000
Visual & hearing disorder	−0.013	−0.013	−0.005	− 0.030	−0.002	0.009	−0.024	− 0.025	−0.056	− 0.084
	0.088	0.145	0.651	0.000	0.626	0.477	0.194	0.046	0.006	0.000
Central nervous disorder	−0.016	−0.014	−0.013	− 0.018	−0.011	− 0.014	−0.057	− 0.055	−0.076	− 0.056
	0.006	0.036	0.100	0.000	0.000	0.140	0.000	0.000	0.000	0.000
malignant tumor	−0.042	−0.063	−0.031	− 0.040	−0.016	0.007	−0.013	− 0.100	−0.055	− 0.191
	0.013	0.001	0.179	0.000	0.028	0.808	0.753	0.000	0.207	0.000
Gastro intestinal disorder	−0.004	−0.017	−0.004	0.002	−0.002	− 0.002	−0.011	− 0.017	−0.029	− 0.054
	0.368	0.001	0.522	0.448	0.296	0.805	0.338	0.025	0.019	0.000
No chronic disease (baseline)										
R^2^	0.024	0.036	0.010	0.052	0.024	0.011	0.021	0.060	0.055	0.112

value represents regression coefficient (upper line) and *p*-value (lower line)

Table 9 shows the Kendall correlations among the HUI3 single-attribute utility scores. A substantial correlation (*r* > 0.25) was only observed for 3 (ambulation and dexterity, speech and cognition, cognition and pain) of the 28 possible comparisons.

**Table 9:** Kendall correlation between HUI3 single attribute score (*n* = 3785)

	HUI3 Attributes							
	Vision	Hearing	Speech	Ambulation	Dexterity	Emotion	Cognition	Pain
HUI3 Attributes								
Vision								
coefficient	1.00	0.06	0.06	0.05	0.05	0.02	0.09	0.09
*p*-value		0.00	0.00	0.00	0.00	0.15	0.00	0.00
Hearing								
coefficient		1.00	0.15	0.20	0.15	0.02	0.08	0.07
*p*-value			0.00	0.00	0.00	0.17	0.00	0.00
Speech								
coefficient			1.00	0.09	0.13	0.15	0.27	0.17
*p*-value				0.00	0.00	0.00	0.00	0.00
Ambulation								
coefficient				1.00	0.46	0.02	0.07	0.14
*p*-value					0.00	0.23	0.00	0.00
Dexterity								
coefficient					1.00	0.04	0.10	0.10
p-value						0.02	0.00	0.00
Emotion								
coefficient						1.00	0.22	0.24
*p*-value							0.00	0.00
Cognition								
coefficient							1.00	0.27
*p*-value								0.00
Pain								
coefficient								1.00
*p*-value								

**Fig. 1:**
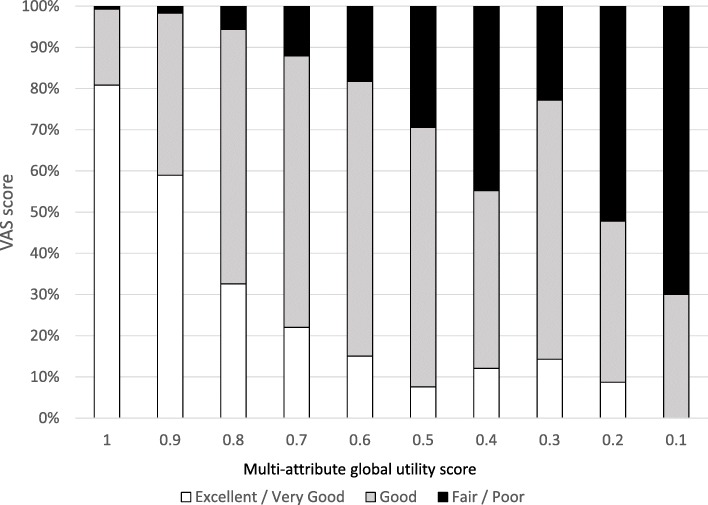
Relationship between multiattribute global HUI3 utility score and self-rated health

Figure 1 shows the relationship between multi-attribute global utility scores and self-rated health. The black area of the bar graph represents the response frequency of “fair” and “poor” regarding self-rated health. The black space decreases gradually as multi-attribute global utility scores increase (except for the group with scores between 0.3 and less than 0.4). Meanwhile, the white area of the bar graph, which represents the response frequency of “excellent” and “very good,” shows a gradual increase as global utility scores increase. Finally, the gray area, which represents “good,” the middle level of self-rated health, remains approximately the same among groups with scores of 0.3 to 0.8 and decreases at both the higher and lower ends of the range of global utility scores.

**Fig. 2:**
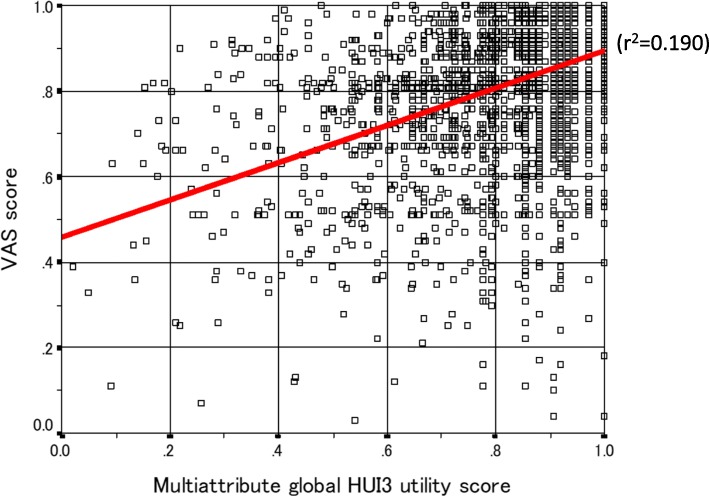
Relationship between multiattribute global HUI3 utility score and VAS score

Figure 2 shows the relationship between global utility scores and VAS scores for all respondents. The correlation coefficient was 0.44, which suggests a moderately positive correlation between the two scores. We also calculated the relationship between HUI3 and VAS score in term of the 10-year age groups; correlation coefficients here were 0.53, 0.52, 0.35, 0.47, 0.29, 0.28, and 0.49, respectively.

## Discussion

For the pilot study featuring the small community sample and a test-retest with a three-week interval, the reliability of the Japanese HUI3 showed high correlation coefficients. Our results suggest that the reliability of the Japanese HUI3 is approximately the same as that of the Canadian version. Boyle et al. [16], examining the Canadian version, reported that for the eight attributes, kappa estimates varied from 0.137 to 0.728 and the interclass correlation for global utility score was 0.767. Considering this, our translation of the HUI3 questionnaire into Japanese seems to have been successful.

In interpreting the results of the community survey, we should note that the age and gender distribution of the sample did not fully represent the general Japanese population. For instance, the community sample was relatively healthy, especially the older age groups, and had a higher household income than the general population.

With respect to personal characteristic variables, the mean global and single-attribute utility scores demonstrated discriminant ability, as expected, but with several exceptions. Notably, especially regarding interpersonal relationships in the workplace and the family and number of chronic diseases, the mean utility scores were lower; furthermore, lower educational level, male gender, being widowed, and seeking work or working part-time were also associated with lower utility scores. This is consistent with the findings of previous HRQOL investigations conducted using generic instruments [31]. However, a recent Japanese population study using the EQ-5D-5 L, EQ-5D-3 L, and SF-6D reported significantly lower scores for female respondents [27], and a large Canadian population survey also reported slightly lower HUI3 global scores for females [20].

Single-attribute utility scores for vision, hearing, speech, cognition, and pain were lower for older age groups, and moreover, as expected, chronic conditions were associated with specific deficits in health status. These results provide initial confirmation of the construct validity of the Japanese HUI3.

This initial confirmation is corroborated by the results of linear regression estimates of factors associated with multi-attribute global utility scores. Controlling for potential confounders, the results revealed a strong relationship between the variables and utility scores, as expected. Almost all negative coefficients were significant between utility score and the variables were significant, and these were hypothesized to reduce HRQOL.

Lower single utility scores were determined to be associated with chronic conditions. Furthermore, as expected, impairments of specific attributes were also associated with chronic conditions. These results are similar to those of Grootendorst et al., who reported evidence of the construct validity of the HUI3 for stroke and arthritis through application of a population health survey in Canada (*n* = 77,663) [20]. Specifically, Grootendorst et al. reported lower global utility scores in respondents with stroke, arthritis, and both, with differences in mean utility scores of − 0.297, − 0.084, and − 0.712, respectively. Respondents with stroke were reported to have lower single-attribute utility scores for speech, ambulation, dexterity, emotion, and cognition. Similarly, the results from the present study show that respondents with musculoskeletal disorder had lower single-attribute utility scores for pain and cognition, while those with central nervous disorders had lower scores for emotion, cognition, and pain.

In the regression results, 7 of the 10 chronic disease categories showed significant negative coefficients for global utility score and, regarding their attributes, significant age-related deterioration. With respect to particular chronic conditions, the expected relationships were in general observed. For instance, significant negative coefficients for emotion and pain were observed for cardiopulmonary disease, and patients with ischemic heart disease do often complain of chest pain and anxiety (risk of sudden death). Meanwhile, negative coefficients for dexterity and pain were observed for musculoskeletal disorder. For central nervous disorder and malignant tumor, there were negative and significant coefficients for five and six of the eight attributes, respectively. Furthermore, negative coefficients for pain were observed for gastrointestinal disorder (perhaps due to stomachache or other abdominal pain). The above observations are notable because in a number of published clinical studies on topics such as pediatric neuro-oncology [32, 33], adult neuro-oncology [33], and survivors of extremely low birth weight [34–36], the use of generic instruments, in particular the HUI, has revealed under-recognized burdens caused by pain; thus, the above findings make a similar contribution by highlighting such a burden among individuals with various chronic diseases. Considering the above, our results provide preliminary evidence that the Japanese HUI3 system has cross-cultural, linguistic, and construct validity.

With respect to the Kendall correlations among the HUI3 single-attribute scores, only 3 of 28 possible cross-comparisons showed substantial correlation (*r* > 0.25). These results suggest acceptable independence among the attributes. This result is also compatible with a report by Houle et al. [37], in which only 2 of 28 comparisons demonstrated substantial correlation.

There was a wide range of global utility scores among respondents who report their health status as “good.” For instance, 30–40% of these respondents had global utility scores lower than 0.4. On the other hand, 60–80% of respondents who reported “excellent” or “very good” had scores higher than 0.8. Similar results were reported by Gold et al., who conducted a survey of 14,407 US adults [29], and in a Canadian survey [37]; however Guertin et al. [20] reported mean global utility scores of 0.942, 0.910, and 0.842 for “excellent,” “very good,” and “good,” respectively, which are higher values than those observed in our survey [20]. VAS scores and global utility scores were positively correlated. Considering the above, the Japanese HUI3 appears to have discriminative validity and interpretability.

Although the reliability, face validity, construct validity, and discriminant validity of the HUI3 have been reported in several studies using the scoring function of the original questionnaire, the question remains as to whether the HUI3 scoring function, which was developed in Canada, can be adopted for Japanese use. Thus, to determine the international generalizability of the HUI3 scoring function, preference surveys of representative and appropriately sized samples of the general populations in Japan should be performed. Then the results should be compared to the results for the ethnically heterogeneous Canadian population that were used when developing the HUI3 scoring function.

Furlong [38], Kaplan [39], Torrance et al. [40], and Feeny et al. [41] reported a substantial heterogeneity among individuals regarding preferences for health states. Furthermore, there is growing evidence that quantitative preferences (values and utilities) are robust when measured with the same procedures, regardless of the population or even the country where the measurement is conducted. For example, the scoring methods for the original Quality of Well-Being scale were developed in the early 1970s based on research with a general population sample from San Diego [42]. When this work was replicated on an arthritic population in the northeast of the United States in the early 1980s, similar results were found [43]. However, many would argue that the population of the US Northeast is culturally different from that of southern California.

Direct support for the international robustness of quantitative preferences measured using the same procedures was provided through the early work of the EuroQol group. This group found that EuroQol VAS scores are similar across three European countries [44]. More recently, LeGales et al. from INSERM replicated the Canadian scoring procedures for the HUI3 in France and obtained quite similar results [45]. Cost-utility analysis using QALYs have been favored in international surveys as patients’ quality of life is especially important, and health care technology must be compared to maximize this [41, 46, 47]. All this is consistent with the growing realization that subjects’ demographic, societal, and cultural characteristics are not consistent predictors of utility; as the common adage states: “poor quality health status is universally recognized and deemed undesirable; this is a constant of being a human being.” In addition, Bosch et al. assessed health status in patients with peripheral arterial disease using the HUI2 and the EuroQol-5D, concluding that the results were very similar even though the HUI2 had been developed in North America and the EQ-5D in Europe [48].

On the other hand, it has long been known that different measurement procedures (e.g., standard gamble, time trade-off, VAS) consistently return differing results; similarly, different multi-attribute systems also produce different results [37, 48]. To clarify this issue, several studies have sought to compare utility scores by applying different multi-attribute instruments to their theoretical models, dimensions, sensitivities, and sources of utility. Brazier et al. reviewed 30 papers describing the mapping (or cross walking) of non-preference-based measures of health to generic preference-based measures. They found the mapping approach to be feasible, but the validity of the models regarding goodness-of-fit and error of prediction at the individual level was highly variable; explanatory power ranged from 0.17 to 0.71, and root mean squared error (RMSE) ranged from 0.084 to 0.2 [49].

Chen et al. also performed mapping between six MAUIs, EQ-5D-5 L, SF-6D, HUI3, 15D, QWB, and AQOL-8D, examining 8022 samples sourced from across six countries. They used four econometric techniques, ordinary least squares, censored least absolute deviations, MM-estimator, and generalized linear model, to show their corresponding predicting powers. For the average HUI3 and HUI3 predicted by the other MAUIs, intraclass correlation ranged from 0.776 to 0.902, while RMSE ranged from 0.1484 to 0.2054 [50].

Using an item response theory analysis, Fryback et al. compared five HRQOL indices, EQ-5D, HUI2, HUI3, QWB-SA, and SF-6D, across a sample of 3844 US adults sourced from the National Health Measurement Study (NHMS). In order to understand the indices’ interrelationships, the researchers combined them into a common scale, consequently finding that EQ-5D, HUI2, and HUI3 are linear with a steep slope over a range from low *θ* (poor health) to the mid-range of *θ*, and then approximately linear with a less steep slope for health below to well above health; however, the inflection points differed for each index, and it was consequently concluded that MAUIs are generally imprecisely related. This may threaten the comparability of evaluations using differing instruments [51].

Although the interpretation of scores using different MAUIs is controversial, such an approach can provide useful information for economic evaluations in which QALYs for which the utility scores have been acquired using different instruments are examined.

The inescapable conclusion regarding the appropriateness of adapting the HUI3 scoring function is that the procedure or instruments matter but the reference population that provides the data does not. The Australian government standardized a small number of instruments similar to the HUI3 and, following a report by Richardson et al. [52], did not recommend that any of these instruments be re-scored for use in Australia. More recently, Richardson et al. [53] compared and explained differences in the magnitude, content, and sensitivities of utilities predicted by the EQ-5D-5 L, SF-6D, HUI3, 15D, QWB, and AQOL-8D. They obtained data from patients from seven disease areas and from healthy individuals from six countries, and reported pairwise linear geometric mean square regression results (such as EQ-5D-5 L = 0.14 + 0.85HUI3 and HUI3 = − 1.074 + 2.09 15D, with *R*^2^ = 0.64 and 0.69, respectively) illustrating the need for transformations between instruments in order to increase their comparability [53].

International comparison of HRQOL and health-adjusted life expectancy has become essential to clarify how differences in levels of socio-economic inequality and health care systems, or access to health care systems over a full life span, affect population health. For instance, Feeny et al. performed a population health comparison between Canada and the US (3505 vs 5183 participants, respectively, white-only population) and found universal health insurance and lower levels of social and economic inequality among the elderly to be influential factors regarding health status [54]. In order to appropriately include non-English-speaking populations in HRQOL comparison, the use of validly translated questionnaires is essential.

Our results indicate that the translation and cultural adaptation of the HUI into Japanese was successful, and we have provided evidence of construct validity and discriminant validity. However, some limitations to this study should be noted.

First, our sample size was not large enough to cover the full range of health states in a population. The sample was generally healthy, wealthy, and highly educated. The age and gender distributions and other sociodemographic factors were not representative of the general Japanese population.

Second, the personal characteristic variables were based solely on self-reports; thus, the results depend on the extent to which the self-reports were accurate.

Third, although the HUI3 scoring function may be quite generalizable in the Western context, the scoring function may not generalize to Japan, where Western culture is not dominant and religious traditions differ.

Thus, considering the above, future studies are needed. Large-scale population health surveys with less selection bias should be conducted to cover a wide range of health statuses and to include a variety of documented clinical conditions. Further surveys, including ones involving cooperation with the Japanese National Livelihood Survey, are necessary to examine the feasibility of the Canadian scoring function for use in Japan.

In spite of the abovementioned study limitations, the Japanese HUI3 appears to be a useful measure of HRQOL in Japan and may be an improvement on standard gamble and time trade-off approaches.

## Conclusion

This study highlights the translation procedures and cultural adaptation of Japanese HUI2 and 3 and measurement properties in a community sample. Translation and adaptation of the HUI3 questionnaire into Japanese was successful, but the sample size and selection bias limit the interpretation of our study conclusions. This study provides evidence of the usefulness of HUI3.

## Data Availability

Data sharing is not applicable to this article as no datasets were generated or analyzed during the current study.

## Abbreviations

ANOVA: Analysis of variance
BMI: Body mass index
CLAD: Censored least absolute deviations
EQ-5D-3 L: EuroQol 5-dimension-3-level
EQ-5D-5 L: EuroQol 5-dimension-5-level
GLM: Generalized linear model
HALE: Health-adjusted life expectancy
HRQOL: Health-related quality of life
HUI3: Health Utilities Index Mark3
ICC: Intraclass correlation
MAUI: Multi-attribute utility instrument
MID: Minimum important difference
OLS: Ordinary least squares
QALY: Quality-Adjusted Life Year
RMSE: Root mean squared error
USD: United States dollars
VAS: Visual analogue scale
